# Sex-related factors influence expression of mood-related genes in the basolateral amygdala differentially depending on age and stress exposure

**DOI:** 10.1186/s13293-016-0106-6

**Published:** 2016-09-17

**Authors:** Rachel Puralewski, Georgia Vasilakis, Marianne L. Seney

**Affiliations:** 1 Department of Psychiatry, University of Pittsburgh, Pittsburgh, PA 15213 USA; 2 Translational Neuroscience Program, University of Pittsburgh, Pittsburgh, PA 15213 USA

## Abstract

**Background:**

Women are twice as likely to be diagnosed with major depressive disorder (MDD) compared to men, but the molecular mechanisms underlying this sex difference are unclear. Previous studies in the human postmortem brain suggest dysfunction in basolateral amygdala (BLA) inhibitory gamma-aminobutyric acid (GABA) signaling and brain-derived neurotrophic factor (BDNF) function, specifically in females with MDD.

**Methods:**

We investigated the effects of sex chromosome complement, developmental gonadal sex, and circulating testosterone on expression of 3 GABA-related and 2 BDNF-related genes in the BLA using three cohorts of four core genotypes (FCG) mice. Cohort 1 included gonadally intact pre-pubertal FCG mice; results were analyzed using two-way ANOVA (sex chromosome complement-by-gonadal sex). We examined the same genes under adult non-stressed (cohort 2) and chronically stressed conditions (cohort 3). The results for cohorts 2 and 3 were analyzed by three-way ANOVA (sex chromosome complement-by-gonadal sex-by-hormone). The use of heatmaps and Spearman correlation of BLA gene expression and anxiety-like behavior provides a global interpretation of gene expression patterns.

**Results:**

In weanlings, we found an effect of sex chromosome complement, with lower expression of GABA/BDNF-related genes in XY mice. Most of these effects did not persist into adulthood, although a number of interesting interactions between organizational and activational effects of hormones emerged. In our adult cohorts, we found that testosterone had different effects depending on stress conditions and/or gonadal sex. Notably, in our chronically stressed adults, we found that the BLA pattern of gene expression for the GABA-related gene, somatostatin (*Sst*), matched the anxiety-like behavior pattern (i.e., lower *Sst* and higher anxiety-like behavior in XY mice, while testosterone increased *Sst* and decreased anxiety-like behavior). Additionally, increased *Sst* gene expression was correlated with decreased anxiety-like behavior.

**Conclusions:**

Sex chromosome complement is an important factor modulating expression of mood-related genes during pre-pubertal development. The observed sex differences under chronically stressed conditions suggest that different molecular profiles may characterize male and female MDD. Our findings here for *Sst* are especially interesting, and suggest an underlying XY vulnerability that is typically compensated for by circulating testosterone in “normal” males. Without testosterone, women may have lower *SST* expression in the amygdala, resulting in increased MDD vulnerability.

## Background

Major depressive disorder (MDD) is a severe and commonly chronic illness characterized by altered mood regulation that is often accompanied by other psychophysiological changes [1]. MDD affects approximately 6.7 % of the US population 18 years or older each year [2], with women being two times more likely than men to be diagnosed with the disorder [3, 4]. Additionally, symptom prevalence also differs between men and women. For instance, women experience more symptoms and have higher severity of symptoms, along with experiencing more subjective distress [5–7]. Women are also more likely to have hyperphagia, hypersomnia, a seasonal effect on mood, and a comorbid anxiety disorder along with their depression [8]. These sex differences suggest that MDD might manifest differently in men and women, with potentially different underlying biological mechanisms driving the disorder. As such, it is important to discern what distinguishes female and male depression in order to not only better understand MDD in each sex, but MDD overall. Eventually, by improving this understanding, we hope to contribute to the goal of developing sex-targeted treatments for depression.

Previous studies provide evidence of dysfunction across corticolimbic brain regions in depressed human subjects, as reviewed in [9, 10]. The corticolimbic circuit is comprised of the dorsolateral prefrontal cortex (DLPFC; an important mood regulation and decision-making brain area), the amygdala (known commonly as the “fear” center), the hippocampus (the memory center), and the subgenual anterior cingulate cortex (sgACC; important for modulation of emotional responses). The DLPFC communicates with the amygdala through the sgACC [11, 12]. As a hub in the corticolimbic network of affect regulation, the amygdala processes emotionally salient stimuli and, in concert with cortical feedback, initiates a behavioral response [13]. Studies using fMRI neuroimaging show that MDD patients exhibit abnormal processing of emotional stimuli, with increased and prolonged amygdala activity in response to a variety of negative stimuli [14, 15] (although [16, 17]). Similarly, amygdala hyperactivity is reported in patients with various anxiety disorders, including post-traumatic stress, generalized anxiety, and social anxiety disorders [18].

Within the corticolimbic circuit, several studies suggest dysfunction in inhibitory gamma-aminobutyric acid (GABA) signaling in MDD. GABA is a key inhibitory neurotransmitter present in each node of the corticolimbic circuit. Studies suggest that decreased GABA inhibition in the circuit contributes to impaired excitation/inhibition balance in mood disorders [19, 20]. Somatostatin (*SST*), a marker of a subtype of GABA interneurons that preferentially target the distal dendrites of excitatory pyramidal cells, is more robustly reduced in female subjects with depression [21]. Interestingly, while *SST* is reduced in the sgACC of both men and women with depression, this *SST* reduction in the amygdala is specific to females, strongly suggesting important potential sex differences in the amygdala of depressed subjects [22]. In addition to the reduction in *SST* in depression, there is also a deficit in expression of the GABA-synthesizing enzymes glutamate decarboxylase 1 (*GAD1*, also known as *GAD67*) and *GAD2* (also known as *GAD65*). Additionally, we previously found tight co-expression of *SST*, *GAD67*, and *GAD65* in the human postmortem brain, suggesting shared function and/or upstream regulators [22]. Taken together, this suggests significant disruption of GABA regulation in subjects with MDD that is specific to females in the amygdala. Brain-derived neurotrophic factor (*BDNF*), which is important for growth and survival of neurons in the brain, is also affected in MDD [23]. Previously, the patterns of gene expression indicate a robust decrease in *BDNF* signaling in depressed subjects (both sexes in the sgACC and females only in the amygdala [23, 24]). TrkB, the BDNF receptor, is also reduced in subjects with MDD [24]. In the amygdala, only females with MDD had a reduction in *BDNF* expression, suggesting that there may be an underlying difference in BDNF-related signaling in the amygdala due to a sex-related factor(s).

We are especially interested in investigating the underlying sex-specific mechanisms that drive the observed sex differences in humans. There are three categories of sex differences we investigate: (1) the effects of sex chromosome complement (driven by XX vs. XY); (2) the effects of developmental gonadal hormones (termed organizational effects of hormones; considered permanent); and (3) the effects of adult circulating hormones (termed activational effects of hormones; considered transient) [25, 26]. While we have observed many sex differences in humans, there are several issues that arise when it comes to identifying clear sex dependent mechanisms underlying these observed human sex differences. First, since sex chromosome complement determines gonadal sex in humans, it is impossible to determine whether observed sex differences are driven by one sex-specific factor over the other. Additionally, we do not know the circulating hormone levels at the time of death for our human postmortem subjects, or how those levels might have varied during human subjects’ lifetimes. These limitations make it difficult to identify specifically which sex-related variables are driving any of the previously observed sex differences in subjects with MDD. With these limitations on human studies, we are motivated to move our studies to mouse models, where we can more tightly control different sex-related factors and more easily manipulate our system of interest [27]. Since the structure of the corticolimbic network in mice is comparable to that of humans, we can study the analogous brain regions in mice as a model for expanding our understanding of the overall circuit.

It is still difficult to discern the three major types of sex differences in typical wild-type mice, as sex chromosome complement still drives gonadal sex. However, we can take advantage of genetically modified strains of mice, such as the four core genotypes (FCG) mice, where sex chromosome complement and gonadal sex are decoupled. By crossing a C57BL/6J female mouse with a XY^−^*Sry* (XYM) male (where the testes-determining gene, *Sry*, is located as an autosomal transgene as opposed to being located on the Y chromosome), we can generate four different genotypes in which sex chromosome complement is no longer tied to gonadal sex [XXF (genetic and gonadal female), XYF (genetic male, gonadal female), XXM (genetic female, gonadal male), and XYM (genetic and gonadal male)]. This allows us to independently analyze the effects of sex chromosome complement and gonadal sex, and better determine which component is responsible for observed sex differences. Moreover, we can manipulate adult circulating hormone levels to examine the activational effects of hormones as well.

In our previous studies examining behavior in chronically stressed FCG mice, we found a decrease in anxiety-like behavior with adult testosterone treatment, as was expected given previous evidence for reduced anxiety with testosterone [28]. However, we also observed a surprising effect of sex chromosome complement, with XY mice having higher anxiety-like behavior compared to XX mice [22]. This was surprising given the higher female prevalence of mood disorders. This led us to speculate that, in a normal male, adult circulating testosterone may override the underlying vulnerability to increased anxiety-like behaviors caused by male sex chromosome complement. It was through the use of FCG mice that the underlying vulnerability in genetic males could be revealed. We were then interested in determining the molecular mechanisms underlying these observed effects on anxiety-like behavior in stressed mice. We began by investigating genes in the frontal cortex as a putative mouse homologue of the sgACC which we have studied in humans with MDD. We found that chronically stressed XY mice had lower expression of *Sst*, *Gad67*, *Gad65*, and *TrkB* compared to chronically stressed XX mice; this gene expression result is completely consistent with XY mice having higher anxiety-like behavior. Additionally, testosterone treatment resulted in reduced frontal cortex *Bdnf* expression, but only in gonadal females [22]. While informative, our findings in the frontal cortex of FCG mice did not fully reflect the behavior pattern we observed, with no consistent effects of testosterone on gene expression despite its potent effect on anxiety-like behavior. We remain interested in these genes due to our findings in the human postmortem brain, but are searching for the node of the corticolimbic network in which these sex-related factors might modulate behavior. Given our findings in humans on sex specificity of *GABA*, *SST*, and *BDNF* deficiency in the amygdala, along with our previous studies on our genes of interest in the frontal cortex of stressed mice, here we chose to examine the amygdala in FCG mice of different ages (adults and pre-pubertal) and stress conditions (non-stressed and chronically stressed) to expand our understanding of how expression of these genes changes during development and under varying stress conditions. Our findings indicate that sex chromosome complement has significant effects on expression of GABA- and BDNF-related genes in weanling and adult stressed mice. Additionally, by focusing our studies on the amygdala, we have revealed a pattern of *Sst* gene expression that reflects the opposing effects of sex chromosome complement and testosterone on anxiety-like behavior. Specifically, XY mice exhibited both lower *Sst* expression and higher anxiety-like behavior compared to XX mice, while testosterone-treated mice had both higher *Sst* expression and lower anxiety-like behavior compared to blank-treated mice.

## Methods

### Mice

The FCG mice used in these studies (originating from Jackson Laboratories, Bar Harbor, ME, USA; B6.Cg-Tg(Sry)2Ei Srydl1Rlb/Arnoj) were generated by crossing a C57BL/6J female with an XY^−^*Sry* male (Y^−^ indicates the absence of *Sry* on the Y chromosome, with *Sry* present instead as an autosomal transgene; XYM). This cross yielded four groups of mice: XX*Sry* males (XXM), XX females (XXF), XY^−^*Sry* males (XYM), and XY^−^ females (XYF). These four genotypes, with gonadal sex determination decoupled from sex chromosome complement, allow us to investigate the effects of sex chromosome complement and developmental gonadal sex (i.e., organizational effects of hormones) independently. The addition of a testosterone or blank capsule (discussed below) after gonadectomy in adulthood also allows us to investigate the effects of circulating hormones (i.e., activational effects of testosterone). The mice were maintained under standard conditions (12/12 h light/dark cycle; 22 ± 1°C, food and water ad libitum), in accordance with the University of Pittsburgh Institutional Animal Care and Use Committee.

### Experimental design

**Fig. 1:**
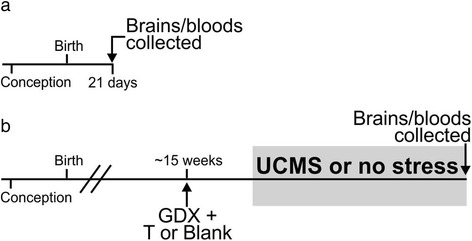
Experimental design for cohorts 1, 2, and 3. **a** Mice in cohort 1 were sacrificed at postnatal day 21 (P21; prior to the onset of puberty). **b** Mice in cohorts 2 and 3 were gonadectomized (GDX) at ~15 weeks of age and implanted subcutaneously with either a testosterone (T)-filled or blank capsule. After GDX, mice in cohort 2 were left unmanipulated until they were sacrificed at 24 weeks of age. After GDX, mice in cohort 3 were exposed to 8 weeks of unpredictable chronic mild stress (UCMS), and then sacrificed (same age as cohort 2). At the time of sacrifice for all cohorts, the brains were harvested for gene expression analyses and bloods were collected for hormone assays

Cohort 1 was comprised of FCG weanlings of each genotype [XXF (*n* = 22), XYF (*n* = 21), XXM (*n* = 21), and XYM (*n* = 17)]. In graphs representing results in this cohort, we show all four groups as well as graphs summarized by main effects [XX (*n* = 43), XY (*n* = 38); F (*n* = 43), M (*n* = 38)]. Cohort 1 mice were sacrificed on postnatal day 21 (P21; with day of birth designated as P0; Fig. 1a). At the time of sacrifice, weanlings were rapidly decapitated, the brains were collected for gene expression studies, and bloods were collected for testosterone assays (described below). Weanlings were neither exposed to stress nor did they receive a circulating hormone manipulation.

Cohort 2 was comprised of non-stressed adult FCG mice housed by gonadal sex and hormone treatment. Additionally, all mice in cohort 2 (Fig. 1b) were gonadectomized at 15 weeks to remove endogenous sources of gonadal hormones; at the time of gonadectomy, half of each genotype was implanted with a subcutaneous testosterone (T)-filled capsule, while the other half of each genotype received a blank (B) capsule [XXF + B (*n* = 11), XXF + T (*n* = 11), XYF + B (*n* = 11), XYF + T (*n* = 10), XXM + B (*n* = 13), XXM + T (*n* = 16), XYM + B (*n* = 13), and XYM + T (*n* = 11)]. In graphs representing results in this cohort, we show all eight groups as well as graphs summarized by main effects [XX (*n* = 51), XY (*n* = 45); F (*n* = 43), M (*n* = 53); B (*n* = 48), T (*n* = 48)]. Cohort 2 did not undergo a stress exposure and was sacrificed at 24 weeks, at which time the brains and bloods were collected.

Cohort 3 was comprised of adult FCG mice (housed by gonadal sex and hormone treatment) that were exposed to unpredictable chronic mild stress (UCMS). Cohort 3 (Fig. 1b) was gonadectomized at 15 weeks, and half of each genotype was implanted with a subcutaneous T-filled capsule while the other half received a blank capsule [XXF + B (*n* = 12), XXF + T (*n* = 18), XYF + B (*n* = 13), XYF + T (*n* = 13), XXM + B (*n* = 18), XXM + T (*n* = 13), XYM + B (*n* = 12), XYM + T (*n* = 13)]. In graphs representing results in this cohort, we show all eight groups as well as graphs summarized by main effects [XX (*n* = 61), XY (*n* = 51); F (*n* = 56), M (*n* = 56); B (*n* = 55), T (*n* = 57)]. After allowing 4 weeks for mice to recover from surgery and for hormone levels to equilibrate, cohort 3 was exposed to 8 weeks of UCMS (details below). Mice were sacrificed at week 24 (while still being exposed to stressors), and the brains and bloods were collected. We previously reported behavioral results and frontal cortex gene expression results from this cohort [22, 29].

### Adult hormone manipulations

Adult FCG mice (cohorts 2 and 3) were bilaterally gonadectomized under isoflurane anesthesia in sterile conditions at 15 weeks of age to remove endogenous source of gonadal hormones. At the time of surgery, mice were implanted with either a subcutaneous silastic (Dow Corning Corp., Midland, MI, USA) capsule containing 5-mm crystalline testosterone (1.57-mm ID × 2.41-mm OD), or with a similarly sized blank capsule. This size testosterone capsule yields circulating testosterone levels at, or slightly above, normal male levels [29]. At the time of sacrifice, trunk blood was collected for testosterone assays (see details below) to verify the efficacy of our adult hormone manipulation.

### Unpredictable chronic mild stress

Mice in cohort 3 were subjected to 8 weeks of UCMS, a behavioral paradigm designed to increase behavioral emotionality and elicit homologous features that are associated with human depression; UCMS respects the timeframe of onset and efficacy of antidepressant treatment [30]. Group-housed mice (gonadal sex and hormone treatment matched) were exposed to a randomized schedule of environmental stressors 7 days a week, gradually increasing in intensity, starting with 1–2 separate stressors a day and ending with 4–5 stressors a day (separately and in tandem with one another) during the final week. Disturbances included light cycle disruption, tilted cage (45° tilt), social stress (rotate mice into previously occupied cages), reduced space (limiting mice to 1/3 of typical space in cage), aversive smell (20 min of exposure to bobcat or fox urine), no bedding or wet bedding overnight, mild restraint (50-mL conical tube with air hole for 15 min), and forced bath (approximately 2 cm of water for 15–45 min). We assessed weight and fur weekly to track progression of the UCMS syndrome.

### Mouse sacrifice and blood collection

Cohort 1 mice (weanlings) were sacrificed by rapid decapitation on postnatal day 21 just as they were weaned from their mothers. The brains were dissected out and immediately flash frozen on dry ice. Trunk blood was collected and allowed to clot at room temperature for 90 min, after which it was spun down to separate the serum from the plasma. The serum was subsequently used in the testosterone hormone assay.

Adult mice (cohorts 2 and 3) were sacrificed at 24 weeks of age. Importantly, mice in cohorts 2 and 3 received the same adult hormone manipulation (i.e., same timing of gonadectomy, same testosterone manipulation, and same amount of time between gonadectomy and sacrifice). Adult mice were anesthetized by isoflurane and rapidly decapitated. The brains were dissected out and immediately flash frozen on dry ice. Trunk blood was collected, allowed to clot at room temperature for 90 min, and the serum separated out to measure testosterone levels.

### Testosterone assay

Circulating testosterone levels of cohort 1 (weanlings) and cohort 2 (adult non-stressed) were measured using a testosterone (mouse/rat) ELISA assay (IBL America; Minneapolis, MN) in accordance with kit directions. Circulating testosterone levels of cohort 3 (adult stressed) were measured offsite with the same ELISA kit, and were previously reported [29]. Importantly, mice treated with testosterone did not differ in testosterone levels and had significantly higher testosterone levels when compared to blank-treated mice (reported below in Results). Additionally, our measured testosterone levels in testosterone-treated mice were within the range of a normal adult male mouse when measured using the same ELISA kit (according to kit product information). Any subjects that fell outside the range for their treatment group (as determined by GraphPad Grubbs’ outlier test) were eliminated from qPCR testing and analysis.

### Processing of brain tissue: BLA dissection and gene expression analyses

Bilateral micropunches (1-mm bore punch) of the BLA (between Bregma −0.94 and −1.82 mm; [31]) were obtained from approximately six 160-μm thick coronal tissue sections cut on a cryostat. All tools were treated with RNase Zap to eliminate any RNases. Punches were stored in RNase free 1.5-mL tubes at −80°C prior to RNA extraction. Total RNA was extracted from BLA tissue punches using RNeasy Plus Kits (Qiagen; Valencia, CA, USA). Cohort 1 (weanling) and cohort 3 (adult stressed) RNA was extracted using RNeasy Plus Micro kits, while cohort 2 (adult non-stressed) RNA was extracted using RNeasy Plus Mini kits with Qiashredder. RNA from the BLA for all cohorts was reverse-transcribed into complementary DNA (cDNA) using QScript cDNA Supermix (olido(dT) and random primers (Quanta Biosciences, Gaithersburg, MD, USA)). Small PCR products were amplified on a MJ Research (Waltham, MA, USA) DNA Engine Opticon System for qPCR using universal PCR conditions (65 to 59 °C touch-down and 40 cycles (10 s at 95 °C, 10 s at 59 °C, and 10 s at 72 °C)). cDNA was amplified in 20-μL reactions (0.1 × SYBR Green, 3 mM MgCl2, 200 nM dNTPs, 200 nM primers, 0.25 unit Platinum Taq DNA Polymerase (Invitrogen, Carlsbad, CA, USA)). Samples were run in triplicate, and results were calculated as the geometric mean of relative intensities compared to three internal controls (actin, glyceraldehyde-3-phosphate dehydrogenase, and cyclophilin). Notably, these internal controls did not differ by sex-related factors, making them acceptable housekeeping genes here. The results are expressed as arbitrary signal (2^−dCT^ × 10,000 [32]). Genes of interest (*Sst*, *Gad67*, *Gad65*, *TrkB*, and *Bdnf*) were selected based on our previous findings in the frontal cortex of FCG mice [22, 29] as well as based on our human postmortem findings [21, 22, 33]. Any gene expression values that were statistical outliers based on Grubbs’ outlier test were excluded from qPCR analysis.

### Heatmap visualization of gene expression results

In order to gain a broader representation of our gene expression results, we created expression heatmaps using matrix2png online software [34]. Expression for each main sex-related factor was calculated by dividing gene expression in the male phenotype by expression in the female phenotype (sex chromosome complement: XY/XX; organizational: testes during development/ovaries during development; activational: testosterone/blank). If expression of the gene was higher in the male phenotype, the color in the heatmap is red; if expression was higher in the female phenotype, the color in the heatmap is blue.

### Correlation between gene expression and anxiety-like behavior

Using Spearman correlation, we compared BLA expression of each gene examined here (*Sst*, *Gad67*, *Gad65*, *TrkB*, *Bdnf*) to anxiety-like behavior in the same cohort of stressed mice (behavior reported separately in [22]). The results from multiple anxiety-like behavior measures were combined into anxiety-like *Z*-scores to reduce the complexity of the correlation analysis (details on *Z*-scores reported in [29]). The anxiety-like behavior measures were compiled from data generated in the elevated plus maze (time in open arms and percent crosses into open arms) and the open field test (time in the center and percent distance traveled in the center). *Z*-scores calculate how many standard deviations (*σ*) an observation (*X*) is above or below the mean of a control group (*μ*).

$$ z-\frac{X-\mu }{\sigma } $$

The first step is to calculate the *Z*-score for each behavioral test measure (e.g., Z_EPM_PercentCrossesOpenArms or Z_OF_PercentTimeCenter); this is performed by normalizing an individual mouse’s test measure to the mean and standard deviation of the comparison group. For all *Z*-scores, we used XX blank mice as the comparison group. The directionality of the *Z*-score was adjusted such that increased values represented increased anxiety-like behavior (e.g., decreased time in the open arms of the elevated plus maze was considered increased anxiety-like behavior). We next calculated the *Z*-score for each mouse per behavioral test by averaging the two individual *Z*-measures for each test (e.g., averaging Z_EPM_TimeOpenArms and Z_EPM_PercentCrossesOpenArms to get Z_EPM_Anxiety). Finally, the overall anxiety-like *Z*-score was calculated by averaging the Z_EPM_Anxiety and Z_OF_Anxiety scores. Correlations were performed between the Z_Anxiety measure for each mouse and expression of each gene (for the entire stressed cohort, as well as performed in groups based on main effects (XX vs. XY, ovaries vs. testes, and blank vs. testosterone treatment)).

### Statistical analysis

Gene expression data for cohort 1 (weanlings) was analyzed using a two-way ANOVA (sex chromosome complement × developmental gonadal sex). Hormone treatment was not included in the ANOVA for cohort 1, as these mice did not receive a hormone manipulation; however, we correlated circulating testosterone levels and gene expression in weanlings using Pearson’s correlation to determine whether endogenous testosterone levels correlated with expression of any gene. Gene expression data for cohorts 2 (adult non-stressed) and 3 (adult stressed) was analyzed using a three-way ANOVA (sex chromosome complement × developmental gonadal sex × hormone treatment). If the ANOVA was significant for any main effect or interaction, we performed planned comparisons using Tukey’s post hoc test. We did not correct for multiple testing in these studies, as we had a priori hypotheses for these genes to be altered based on our findings in the frontal cortex of FCG mice and in human postmortem brain tissue of patients with MDD [21–24, 29]. Data are expressed as mean ± SEM, and significance was set at *p* < 0.05.

## Results

### FCG weanlings (cohort 1)

**Fig. 2:**
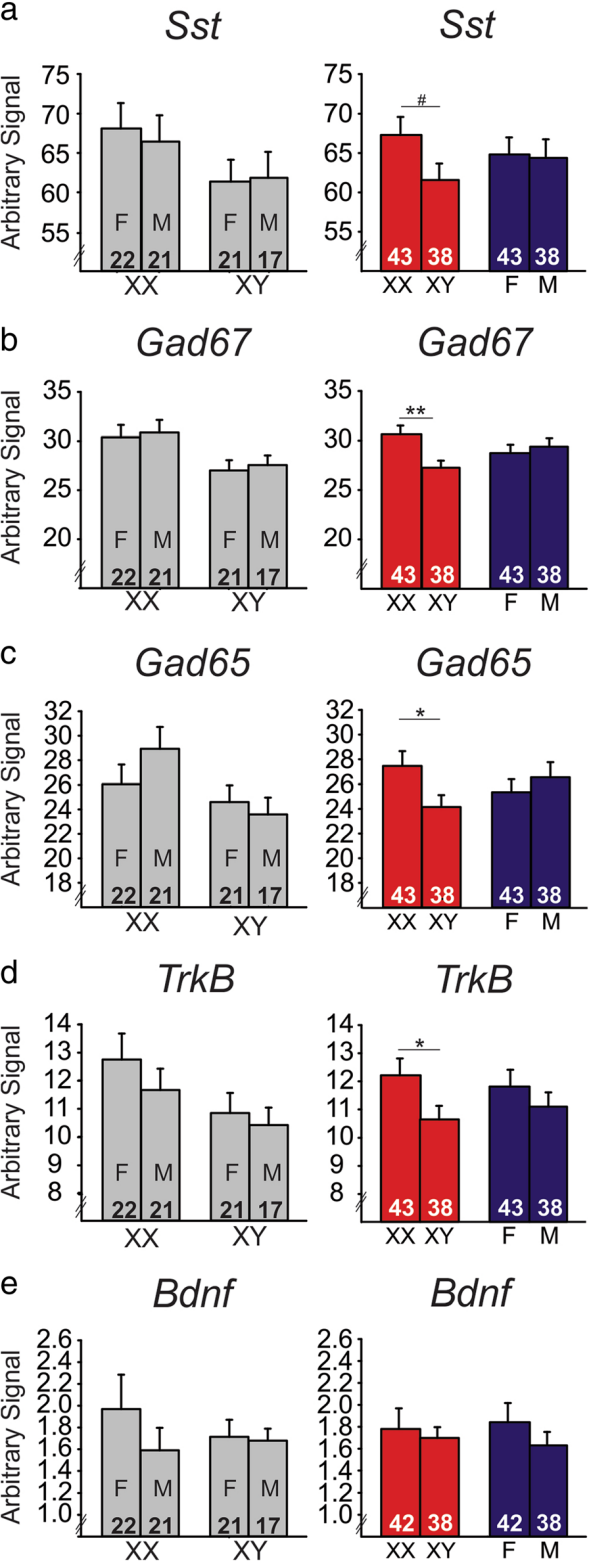
The effects of sex-related factors on expression of GABA- and BDNF-related genes in the basolateral amygdala of weanling mice. Mice with male sex chromosome complement (XY) had significantly lower expression of *Sst* (**a**), *Gad67* (**b**), *Gad65* (**c**), and *TrkB* (**d**) compared to XX mice; there was no effect of sex chromosome complement on *Bdnf* expression (**e**). There were no organizational effects of hormones on expression of any gene investigated (**a**–**e**). For each gene, *graphs* on the *left* show results individually for all four experimental groups. *Graphs* on the *right* summarize the same data as shown on the left after collapsing across main effects. *Numbers* at base of bars indicate *N*. For main effect comparisons: **p < 0.01; *p < 0.05; ^#^ *p* < 0.1. *F* gonadal female, *M* gonadal male

Gonadally intact FCG mice (XXF, XXM, XYF, XYM) were sacrificed at the time of weaning (P21, Fig. 1a); this time-point was selected to precede the onset of puberty. We then examined the effects of sex chromosome complement and gonadal sex (i.e., organizational effects of hormones) on expression of several genes in the BLA. There was a main effect of sex chromosome complement, with XY mice having lower expression of *Sst* (*p* < 0.08; Fig. 2a), *Gad67* (*p* < 0.01; Fig. 2b), and *Gad65* (*p* < 0.035; Fig. 2c), as well as *TrkB* (*p* < 0.05; Fig. 2d) in the BLA compared to XX mice. There was no effect of sex chromosome complement on *Bdnf* expression in the BLA (Fig. 2e, *p* > 0.1). Compared to gonadal females, gonadal males showed no significant difference in expression of *Sst* (Fig. 2a), *Gad67* (Fig. 2b), *Gad65* (Fig. 2c), *TrkB* (Fig. 2d), or *Bdnf* (Fig. 2e) in the BLA (*p* > 0.3 for all comparisons). At the time of sacrifice, we also collected blood serum to measure circulating testosterone levels. Mice in cohort 1 did not receive artificial hormone treatment, and did not differ in circulating testosterone levels among genotypes (XX 0.127 ng/mL ± 0.008; XX*Sry* 0.137 ng/mL ± 0.010; XY^−^ 0.133 ng/mL ± 0.010; XY^−^*Sry* 0.139 ng/mL ± 0.011; *p* > 0.4). Seven subjects were eliminated due to testosterone levels outside of the expected normal circulating hormone levels (>0.25 ng/mL). All of these subjects were gonadally male (5 XXM, 2 XYM). Even when these seven subjects were included in the gene expression analyses, we found that our results remained consistent (i.e., same genes were significant and same genes were not significant; data not shown), indicating that the circulating testosterone levels did not have an effect on gene expression. Additionally, there were no significant differences in circulating testosterone across sex chromosome complement (XX 0.131 ng/mL ± 0.04; XY 0.140 ng/mL ± 0.05; *p* > 0.3) or gonadal sex (M 0.129 ng/mL ± 0.04; F 0.143 ng/mL ± 0.05; *p* > 0.1) when the outliers were removed from analysis. When the seven subjects were included, we found that testosterone was significantly higher in gonadal males than in gonadal females (*p* < 0.03); however, this is expected considering that the eliminated subjects were all gonadally male with slightly higher testosterone levels. Importantly, these results confirm that testosterone levels at P21 did not differ between XXM and XYM mice (XXM 0.137 ng/mL ± 0.01; XYM 0.139 ng/mL ± 0.011; *p* > 0.45). Additionally, circulating testosterone did not correlate with expression levels of any gene of interest (*p* > 0.5 for all correlation analyses). Together, this suggests that any difference in gene expression we might observe based on gonadal sex in weanlings will be due to organizational effects of hormones rather than activational effects of testosterone.

### FCG non-stressed adults (cohort 2)

**Fig. 3:**
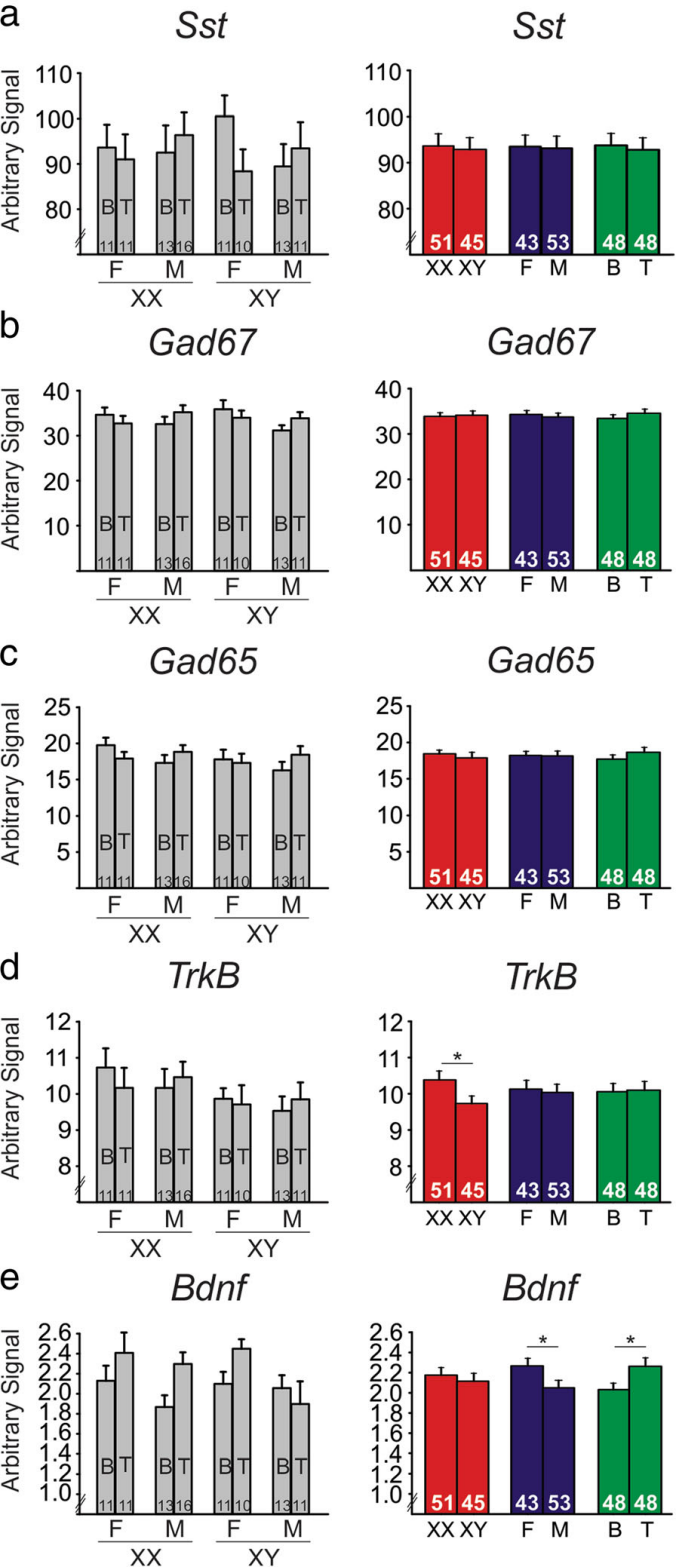
The effects of sex-related factors on expression of GABA- and BDNF-related genes in the basolateral amygdala of non-stressed adult mice. There was no effect of sex chromosome complement on expression of *Sst* (**a**), *Gad67* (**b**), *Gad65* (**c**), or *Bdnf* (**e**). However, mice with male sex chromosome complement (XY) had significantly lower expression of *TrkB* (**d**). **e** Mice with male developmental gonadal sex had lower *Bdnf* expression compared to mice with female developmental gonadal sex. Testosterone significantly increased expression of *Bdnf*. For each gene, *graphs* on the *left* show results individually for all eight experimental groups. *Graphs* on the *right* summarize the same data as shown on the left after collapsing across main effects. *Numbers* at the base of bars indicate *N*. For main effect comparisons: *p < 0.05. *T* testosterone, *B* blank, *F* gonadal female, *M* gonadal male

In cohort 2 (Fig. 1b), FCG mice were gonadectomized in adulthood to allow typical gonadal hormone exposure during critical periods of development. Thus, any significant effects of gonadal sex here are indicative of organizational effects of hormones during development. At the time of gonadectomy, mice were given a hormone replacement in the form of a blank- or testosterone-filled capsule to examine the activational effects of circulating testosterone. In cohort 2, mice treated with testosterone capsules did not differ in serum testosterone levels (*p* > 0.1). Additionally, testosterone-treated cohort 2 mice had significantly higher testosterone levels compared to blank-treated mice (blank 0.1425 ng/mL ± 0.0115; testosterone 2.802 ng/mL ± 0.1341; *p* < 0.001). Mice were sacrificed 12 weeks after gonadectomy; mice in cohort 2 were not exposed to chronic stress in adulthood (in contrast to cohort 3). We then examined main effects of sex chromosome complement, gonadal sex, and hormone treatment, along with any interactions between main effects on BLA gene expression. We did not observe a significant effect of sex chromosome complement on *Sst* (Fig. 3a), *Gad67* (Fig. 3b), *Gad65* (Fig. 3c), or *Bdnf* (Fig. 3e) expression in adult non-stressed mice (*p* > 0.5 for all comparisons). However, similar to what was observed in weanlings, adult non-stressed XY mice had significantly lower expression of *TrkB* compared to XX mice (*p* = 0.05, Fig. 3d). In contrast with what we found in weanlings, we observed a significant effect of developmental gonadal sex (i.e., organizational effect of hormones) on *Bdnf* expression in the BLA (Fig. 3e). Specifically, mice with testes during development had significantly lower expression of *Bdnf* compared to mice that had ovaries during development (*p* < 0.025). There was no significant effect of developmental gonadal sex on BLA expression of *Sst* (Fig. 3a), *Gad67* (Fig. 3b), *Gad65* (Fig. 3c), or *TrkB* (Fig. 3d; *p* > 0.6 for all comparisons). There was also a significant activational effect of testosterone on *Bdnf* expression, with testosterone-treated mice having higher BLA *Bdnf* expression than blank-treated mice (*p* < 0.035, Fig. 3e). There was no activational effect of testosterone on *Sst* (Fig. 3a), *Gad67* (Fig. 3b), *Gad65* (Fig. 3c), or *TrkB* (Fig. 3d; *p* > 0.3 for all comparisons).

**Fig. 4:**
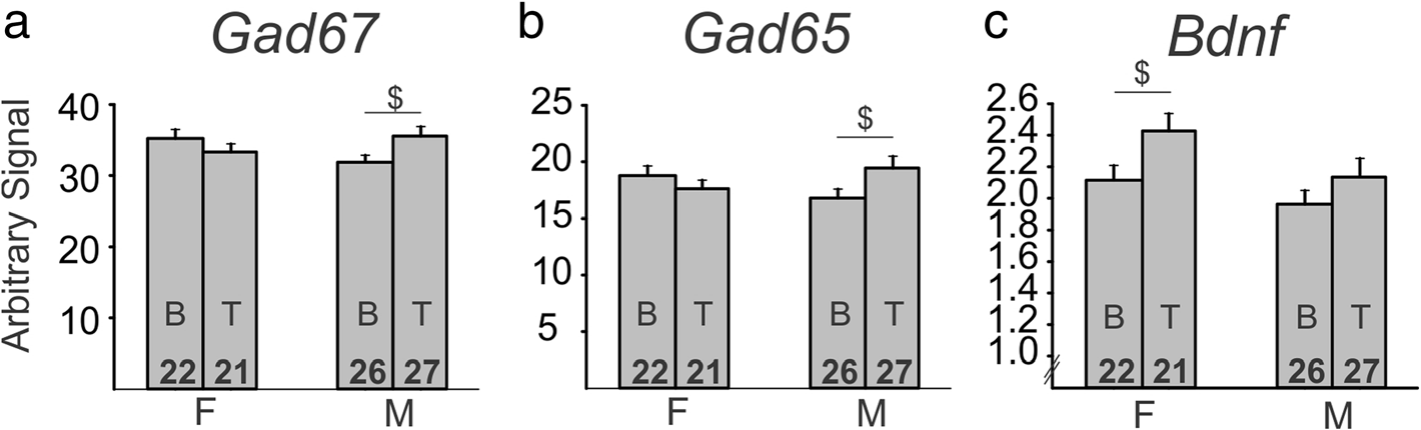
Interactions of sex-related factors on expression of GABA- and BDNF-related genes in the basolateral amygdala of non-stressed adult mice. There was a significant gonadal sex by hormone interaction on expression of *Gad67* (**a**) and *Gad65* (**b**), with testosterone significantly increasing gene expression only in gonadal males. **c** There was a significant gonadal sex by hormone interaction for *Bdnf*, with testosterone significantly increasing expression only in gonadal females. *Numbers* at the base of bars indicate *N*. For post hoc two-group comparisons: ^*$*^ *p* < 0.05. *T* testosterone, *B* blank, *F* gonadal female, *M* gonadal male

In addition to investigating the main effects in the non-stressed adult cohort, we also found a number of interesting interactions of main effects on BLA gene expression. We found a significant gonadal sex by hormone interaction for both *Gad67* (*p* < 0.025, Fig. 4a) and *Gad65* (*p* < 0.032, Fig. 4b). *Gad67* and *Gad65* exhibited similar patterns of gene expression, with testosterone having no effect on gonadal females but testosterone increased expression of both genes in gonadal males (*p* < 0.05 for both genes). In another exploratory analysis, we found that testosterone increased *Bdnf* expression in gonadal females (*p* < 0.037), but had no effect on *Bdnf* expression in gonadal males (*p* > 0.2; Fig. 4c).

### FCG stressed adults (cohort 3)

**Fig. 5:**
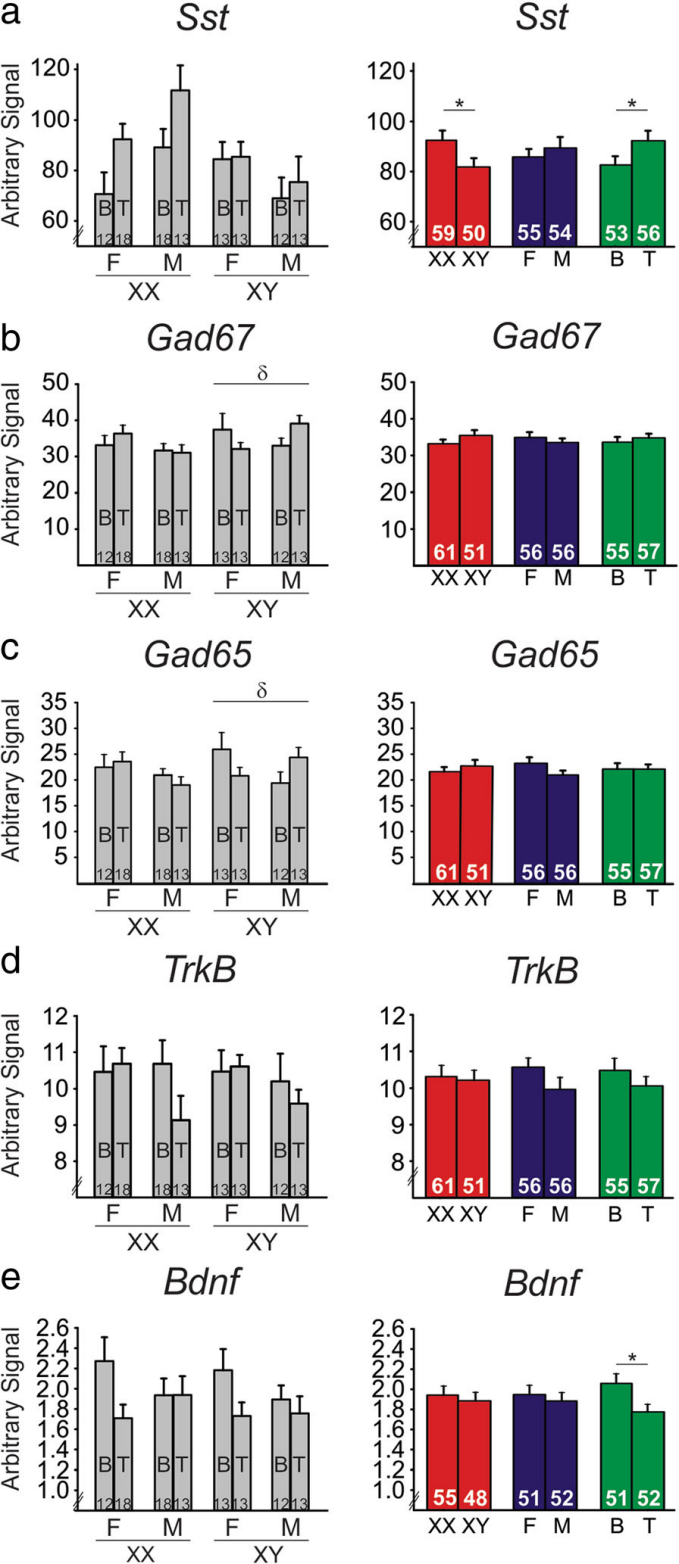
The effects of sex-related factors on expression of GABA- and BDNF-related genes in the basolateral amygdala of stressed adult mice. **a** Mice with male sex chromosome complement (XY) had lower expression of *Sst*. Additionally, mice treated with testosterone had higher *Sst* expression. There were no main effects of sex chromosome complement on expression of *Gad67* (**b**), *Gad65* (**c**), *TrkB* (**d**), or *Bdnf* (**e**). There were no main effects of gonadal sex on gene expression (**a**–**e**). Testosterone significantly decreased expression of *Bdnf* (**e**), but did not affect expression of *Gad67* (**b**), *Gad65* (**c**), or *TrkB* (**d**). There was a significant three-way sex chromosome complement × gonadal sex × hormone interaction on expression of *Gad67* (**b**, *left*) and *Gad65* (**c**, *left*). Specifically, the post hoc analyses revealed a significant interaction of gonadal sex and hormone only in XY mice. *Graphs* on the *left* show results individually for all eight experimental groups. For each gene, *graphs* on the *right* summarize the same data as shown on the left after collapsing across main effects. *Numbers* at the base of the bars indicate *N*. For main effect comparisons: *p < 0.05. For interaction between gonadal sex and hormone: ^*δ*^ *p* < 0.05. *T* testosterone, *B* blank, *F* gonadal female, *M* gonadal male

In cohort 3, an additional set of FCG mice was gonadectomized and given testosterone or blank capsules at 15-week old (same age as non-stressed adults in cohort 2, Fig. 1b). However, mice in cohort 3 were additionally subjected to 8 weeks of UCMS to induce elevated anxiety-/depressive-like behaviors. Compared to XX mice, XY mice had significantly lower *Sst* expression (*p* < 0.04; Fig. 5a); this pattern is similar to what we observed in weanlings (Fig. 2a). There was no effect of sex chromosome complement on expression of *Gad67* (Fig. 5b), *Gad65* (Fig. 5c), *TrkB* (Fig. 5d), or *Bdnf* (Fig. 5e) in the BLA (*p* > 0.1 for all comparisons). Compared to mice with ovaries during development, mice with testes during development showed no significant differences in expression of *Sst* (Fig. 5a), *Gad67* (Fig. 5b), *Gad65* (Fig. 5c), *TrkB* (Fig. 5d), or *Bdnf* (Fig. 5e) in the BLA (*p* > 0.1 for all comparisons). There was a main activational effect of testosterone exposure on *Sst*, with testosterone increasing *Sst* expression (*p* < 0.033, Fig. 5a). Similar to what was observed in cohort 2 (adult non-stressed), there was a main effect of circulating testosterone on BLA *Bdnf* expression (*p* < 0.023, Fig. 5e). Interestingly, however, this difference occurred in the opposite direction as observed in the non-stressed cohort (compare Figs. 3e and 5e), with testosterone decreasing BLA *Bdnf* expression in stressed mice. There were no significant effects of testosterone on expression of *Gad67* (Fig. 5b), *Gad65* (Fig. 5c), or *TrkB* (Fig. 5d) expression in the BLA (*p* > 0.2 for all comparisons).

**Fig. 6:**
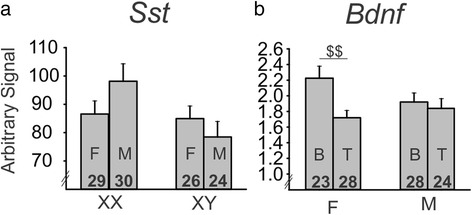
Interactions of sex-related factors on expression of GABA- and BDNF-related genes in the basolateral amygdala of stressed adult mice. **a** There was a significant sex chromosome complement by gonadal sex interaction for *Sst*, with a pattern for an increase in expression in gonadal males compared to gonadal females for XX mice and a pattern of a decrease in expression in gonadal males compared to gonadal females for XY mice. **b** There was a trend for an interaction of gonadal sex and hormone on *Bdnf* expression, with exploratory analysis revealing that testosterone decreased *Bdnf* expression only in gonadal females. *Numbers* at the base of the bars indicate *N*. For post hoc two-group comparisons: ^*$$*^ *p* < 0.01. *T* testosterone, *B* blank, *F* gonadal female, *M* gonadal male

We also investigated a number of interactions between the main effects. We found a significant sex chromosome complement by gonadal sex interaction for *Sst* expression in the BLA (*p* < 0.037, Fig. 6a). We also found a trend for a gonadal sex by hormone interaction on *Bdnf* expression (*p* < 0.082; Fig. 6b). Exploratory follow-up analyses revealed that testosterone treatment decreased *Bdnf* expression in gonadal females (*p* < 0.01), but had no effect on gonadal males (*p* > 0.1). Again, this difference in *Bdnf* expression was the opposite of what was observed in non-stressed mice in cohort 2 (compare Figs. 4c and 6b). In contrast to what we observed in cohort 2 (non-stressed adults), the interaction of gonadal sex and testosterone treatment was not significant for *Gad67* or *Gad65* (*p* > 0.1). However, the three-way interaction of sex chromosome complement, gonadal sex, and testosterone treatment was significant for both *Gad67* (*p* < 0.039, Fig. 5b, left) and G*ad65* (*p* < 0.027, Fig. 5c, left). Specifically, there was a significant gonadal sex by hormone treatment interaction in XY, but not in XX mice. When the data for *Gad67* and *Gad65* gene expression is split by sex chromosome complement, it becomes evident that the pattern of gene expression observed in the non-stressed cohort (Fig. 4a, b) is reflected in stressed XY mice, but not in stressed XX mice. Specifically, the gonadal by hormone interaction in stressed XY mice is significant for *Gad67* (*p* < 0.05, Fig. 5b, left) as well as *Gad65* (*p* < 0.035, Fig. 5c, left), while the gonadal by hormone interaction in XX mice is not significant (*p* > 0.1).

### Heatmap visualization of gene expression by main sex-related factors

**Fig. 7:**
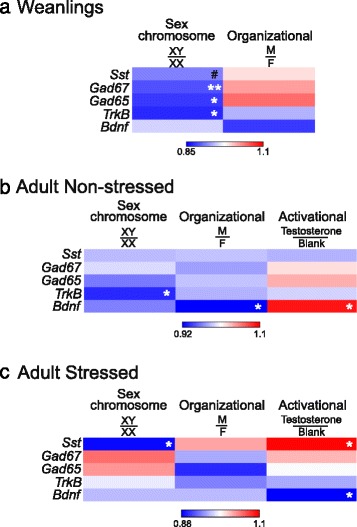
Heatmap representation of basolateral amygdala gene expression results. Expression for each main factor is expressed as the “male” phenotype divided by the “female” phenotype (i.e., sex chromosome complement, XY/XX; organizational, male gonads/female gonads; activational, testosterone/blank). *Red* is indicative of higher levels of expression in male phenotypes; *blue* is indicative of higher levels of expression in female phenotypes. Heatmap representation was done for **a** weanlings, **b** adult non-stressed, and **c** adult stressed. For main effect comparisons: **p < 0.01; *p < 0.05; ^*#*^ *p* < 0.1

In order to summarize our gene expression results and reveal patterns of influence for sex-related factors on BLA gene expression, we visualized our results using heatmaps. As was evident from the qPCR data, there was a pattern for significantly lower expression of *Sst*, *Gad67*, *Gad65*, and *TrkB* in XY weanling mice compared to XX weanling mice (Fig. 7a). Similarly, this pattern was reflected in the adult non-stressed cohort (Fig. 7b), although only statistically significant for *TrkB*. This pattern was not reflected overall in the adult stressed mice (Fig. 7c), except for *Sst* and *Bdnf*. The heatmap revealed different patterns of expression when examining organizational effects of hormones (i.e., gonadal) on gene expression. In weanlings, with no significant differences between groups, we observed a pattern for higher expression of *Gad67* and *Gad65* in gonadal males, as well as a pattern for lower expression of *TrkB* and *Bdnf* in gonadal males (Fig. 7a). Interestingly, this pattern changed in the adult non-stressed cohort to reflect lower expression for all genes in mice with testes during development (Fig. 7b). This general pattern was present in the adult stressed cohort as well (Fig. 7c). Finally, we used the heatmaps to compare activational effects of testosterone on expression of genes in adult mice. There was no clear pattern of expression in adult non-stressed mice across genes (Fig. 7b). However, the heatmap for cohort 3 revealed that both *Sst* and *Bdnf* had the opposite pattern of expression under stressed conditions compared to non-stressed conditions. *Sst* now showed significantly higher expression with testosterone treatment while *Bdnf* showed significantly lower expression (Fig. 7c). Together, these results indicate complicated balancing roles between sex-related factors, which differ based on age and stress exposure.

### Correlation between gene expression and anxiety-like behavior

Refer to Table 1 for statistical information associated with the correlation analysis. We first examined the data for significant correlations between anxiety-like behavior and gene expression in all mice subjected to UCMS. We did not perform behavior testing in the weanling or adult non-stressed cohorts, as we did not want these cohorts to be exposed to the stress of behavior testing. We found that *Sst* was negatively correlated with anxiety-like behavior (*p* = 0.041) for the entire stressed cohort (i.e., higher *Sst* expression levels were correlated with decreased anxiety-like behavior). There was also a trend (*p* = 0.077) for a positive correlation between *Bdnf* expression and anxiety-like behavior for the entire stressed cohort (i.e., higher *Bdnf* expression levels were correlated with increased anxiety-like behavior). There were no other correlations between anxiety-like behavior and expression of the remaining genes (*Gad67*, *Gad65*, *TrkB*; *p* > 0.25 for all comparisons) in the entire stressed cohort. We then split the cohort into groups based on each main sex-related factor (XX, XY, gonadal males, gonadal females, blank-treated, testosterone-treated) and again performed correlation analyses between gene expression and anxiety-like behavior. We found a trend for *Sst* to be negatively correlated with anxiety-like behavior in gonadal males (*p* = 0.089), with higher *Sst* expression levels being correlated with decreased anxiety-like behavior. We also found a positive correlation between *Bdnf* expression and anxiety-like behavior (*p* = 0.02) in XY mice and a trend (*p* = 0.058) in gonadal females (i.e., higher *Bdnf* expression levels were correlated with increased anxiety-like behavior). We found no other correlations between gene expression and anxiety-like behavior when the cohort was split by main sex-related variables (*p* > 0.1 for all comparisons).

**Table 1:**
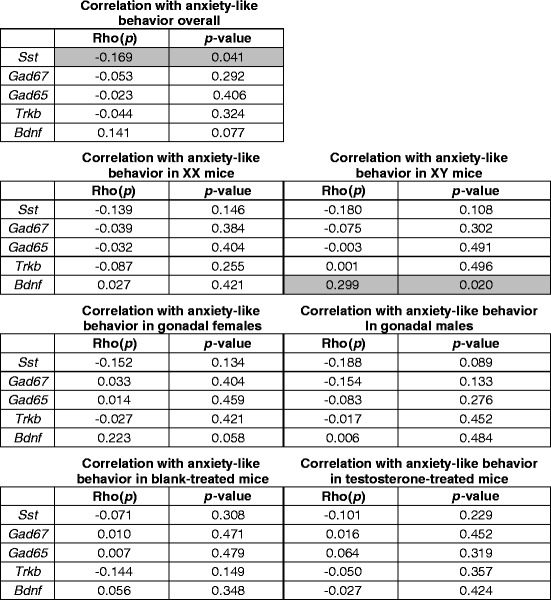
Correlation between basolateral amygdala gene expression and anxiety-like behavior

Gray shades indicate significant correlations (*p* < 0.05)

## Discussion

Through examination of gene expression in the BLA of FCG mice at multiple developmental time-points and under different stress conditions, we were able to reveal several sex-based differences in expression of GABA-related and BDNF-related genes. In weanlings (Fig. 2), sex chromosome complement significantly influenced expression of *Sst*, *Gad67*, *Gad65*, and *TrkB*, with lower expression of these genes in XY compared to XX mice. Gonadal sex and circulating testosterone did not influence expression of these genes in weanlings. In non-stressed adult mice (Fig. 3), *TrkB* continued to be significantly different based on sex chromosome complement (XX > XY). *Bdnf* expression was also influenced by sex-related factors in non-stressed adult mice, with opposing effects of developmental gonadal sex (gonadal female > gonadal male) and male-like testosterone levels (blank < testosterone). Organizational and activational hormone interactions for *Gad67*, *Gad65*, and *Bdnf* highlight the distinct role that circulating hormones such as testosterone play depending on gonadal sex. In stressed adult mice (Fig. 5), we found opposing effects of sex chromosome complement (XX > XY) and male-like testosterone levels (blank < testosterone) on *Sst* expression in the BLA. *Bdnf* expression continued to be affected by hormone treatment in adult stressed mice, but the effect was opposite to what we observed in non-stressed adults (blank > testosterone).

Exposure to stress and trauma during adolescence can have long-term effects on neural transmission and disease vulnerability. Given this, the implications of our weanling results are especially of interest to us. The development of the GABA system in particular has many long-term effects on the structure and communication of the cortex as a whole. GABA initially begins as an excitatory neurotransmitter following birth, but continues to develop during the pre-pubertal stages and becomes inhibitory (reviewed in [35]). This transition is significant, as it contributes to synaptic development that occurs during puberty and adulthood. As the GABA system develops during adolescence, it also contributes to the synchronization of cortical activity (reviewed in [36]). Dysregulation of cortical synchronization is thought to be a possible contributor to the development of MDD, with increased synchronization of neuron firing observed in subjects with the disorder [37]. Differences in GABA function between males and females during adolescence may influence sex-specific synaptic development or cortical synchronization later in life, subsequently affecting cortical development and vulnerability for psychiatric diseases such as MDD. Here, we found that weanling mice with XY sex chromosome complement had lower GABA-related gene expression compared to mice with XX sex chromosome complement. Given that adult XY mice show elevated anxiety-like behavior in adulthood [22], this pre-pubertal difference in GABA-related gene expression may contribute to adult anxiety-/depressive-like behavior. Differences in GABA-related gene expression based on sex chromosome complement in weanlings could permanently affect the way the brain communicates by affecting how neurons signal one another and are synchronized in adulthood. Perhaps more importantly, the pre-pubertal results emphasize that early amygdala development differs significantly by sex chromosome complement and deserves more attention given the long-lasting effects these GABA differences could have, with potentially different developmental trajectories in XY and XX mice.

The lack of organizational effects of hormones in weanlings becomes more interesting when examined within the context of all three tested cohorts. Since we observed sex differences in gene expression due to organizational effects of hormones in both of our adult cohorts, while not observing any sex differences in our pre-pubertal mice, this suggests that the observed sex differences are due to effects of hormones from the gonads during puberty. This holds important implications for understanding the role gonads during puberty play in affecting not only behavior (as reviewed in [38]) but also in the development of the cortex (as reviewed in [38],[39]). Since these sex differences in gene expression are observed in our adult cohorts, this further suggests that these organizational effects that occur during puberty drive permanent effects on gene expression in the BLA. This finding is also interesting since puberty is the developmental time-point when the sex difference in depression incidence emerges (e.g., [40–44]).

Although we did not compare our non-stressed and stressed cohorts directly within the same experiment, we did find distinct patterns of gene expression within each stress exposure. Some patterns of gene expression only emerged when adult mice were exposed to stress. For instance, the opposing effects of male sex chromosome complement and testosterone on *Sst* expression (i.e., lower *Sst* expression in XY mice, but higher expression in mice treated with testosterone) only emerged under stressed conditions. Similarly, testosterone had an opposite effect on *Bdnf* expression based on whether the mice were chronically stressed or not, with testosterone increasing *Bdnf* expression under non-stressed conditions, but decreasing expression under chronic stress conditions. Likewise, some sex-related effects are masked under chronic stress conditions. For instance, the effect of sex chromosome complement on *TrkB* disappears under chronic stress conditions, along with the organizational effect on *Bdnf* expression. With these changes in patterns of gene expression, we can begin to construct our interpretation of the GABA and BDNF systems function under no stress and chronically stressed conditions.

Here, we found that XX mice had higher *TrkB* expression compared to XY mice in weanlings as well as in non-stressed adults. Interestingly, this difference based on sex chromosome complement did not persist under adult stressed conditions. Previously, we found that frontal cortex *TrkB* expression was negatively correlated with anxiety-like behavior [29]. This evidence supports a potential underlying XY vulnerability for MDD that is reflected in changes to the BDNF system. In addition to the changes observed in *TrkB*, *Bdnf* also shows changes in its pattern of expression due to sex-related factors. Interestingly, these differences are dependent on stress exposure. Specifically, we found that testosterone increased *Bdnf* expression in non-stressed gonadal females, but decreased *Bdnf* expression in stressed gonadal females. Another study found higher *Bdnf* expression in the BLA of gonadally intact adult male mice compared to female mice [45]. Here, we observed an increase in *Bdnf* expression in non-stressed mice treated with testosterone, even in those that were gonadally female. This suggests that the sex difference in BLA *Bdnf* expression reported in [45] may be due to circulating testosterone, as opposed to organizational effects of hormones or sex chromosome complement. It is important to emphasize that this is an example of how circulating hormones can affect gene expression differently depending on stress conditions, and also depending on other sex-related factors. Overall, these changes to the *Bdnf* system hold important implications about neural communication in the brain. As *Bdnf* works largely to maintain levels of neuronal excitation through inhibition of GABA signaling, decreases in *Bdnf* or *TrkB* may contribute to an increase in GABA inhibitory signaling. Moreover, testosterone decreased both anxiety-like behavior and *Bdnf* expression in stressed mice, and there was a trend for a positive correlation between *Bdnf* expression and anxiety-like behavior. Together, this suggests that the anti-anxiety effects of testosterone might be mediated by changes in *Bdnf*. When we split the cohort into groups based on sex-related factors, the positive correlation between anxiety-like behavior and *Bdnf* expression was found in XY mice and in gonadal females only, suggesting that the effects of *Bdnf* expression on anxiety-like behavior are more salient in XY mice and in gonadal females.

*Gad67* and *Gad65* consistently show the same expression patterns within each experimental condition. This provides further support that the two are co-regulated/co-expressed and behave similarly under a variety of stress and age conditions, as well as with various sex-related factors. It is important to note, however, that within each age or stress exposure, the sex-related factors affected *Gad67* and *Gad65* expression differently. For instance, there was a significant interaction between gonadal sex and testosterone treatment on *Gad67* and *Gad65* expression in non-stressed adults, but a significant three-way interaction between sex chromosome complement, gonadal sex, and testosterone in stressed adults. Specifically, the interaction between gonadal sex and testosterone is reflected only in XY mice under stressed conditions. It is possible that stress exposure exacerbates changes in the GABA system only in XY mice. Since we observed an effect of sex chromosome complement on *Gad67* and *Gad65* expression pre-pubertally and in stressed adults, but not in non-stressed adults, this suggests a possible difference in the pre-pubertal condition that is only revealed again in adulthood when an animal is stressed.

We previously found that mice with XY sex chromosome complement had lower *Sst* expression in the frontal cortex along with higher anxiety-like behavior compared to XX mice [22]. However, in the frontal cortex, testosterone did not influence *Sst* expression, despite the potent effect of testosterone to decrease anxiety-like behavior. The pattern of *Sst* gene expression we observed in the BLA (lower *Sst* levels in XY mice, with testosterone increasing *Sst* expression) is consistent with the previous behavior results. Specifically, lower *Sst* expression in the BLA with XY sex chromosome complement is accompanied by higher anxiety-like behavior, while an increase in *Sst* expression with testosterone is accompanied by a decrease in anxiety-like behavior. Further, our correlation analysis revealed a significant negative correlation between *Sst* expression and anxiety-like behavior (i.e., higher *Sst* expression was correlated with decreased anxiety-like behavior). When split by main sex-related factors, we found a trend for a negative correlation only in gonadal males, seeming to imply that developmental gonadal sex may have important effects on changes in *Sst* along with behavior. Having previously found *SST* to be reduced in the sgACC and DLPFC of both males and females with MDD, but only in females with MDD in the BLA, it follows that *SST* action in the BLA may be contributing to female vulnerability to MDD. Our results here also show that *Sst* expression is increased by testosterone, which also decreases anxiety-like behavior. Given that females do not typically have high levels of circulating testosterone, this may be influencing amygdala *SST* levels in females, also potentially contributing to female MDD vulnerability.

Our results for *Sst* suggest that sex chromosome complement and testosterone may be having conflicting actions on BLA gene expression to modulate behavior. This “compensation” phenomena has been described in the literature, with the effects of male sex chromosome complement counteracting effects of testosterone (or vice versa), serving to decrease male-female differences [46]. Other studies have found similar patterns of results. For instance, while testosterone increases male sex behavior, XY mice exhibit lower male sex behavior compared to XX mice [47].

Our results indicating that testosterone both decreases anxiety-like behavior [22] and increases expression of GABA-related genes in the BLA are consistent with findings in both humans and rodents. For instance, XY humans with complete androgen insensitivity syndrome exhibit elevated levels of mood disorders [48–52], suggesting a protective role for testosterone related to mood. Additionally, low testosterone in human males is associated with higher anxiety, and testosterone treatment results in reduced anxiety in these men [53, 54]. Several studies in rodents report similar anti-anxiety effects of testosterone. For instance, rats with the testicular feminization mutation (Tfm) of the androgen receptor exhibit higher anxiety-like behaviors compared to wild-type males and females [55]. Additionally, mice with either a spontaneous or induced Tfm of the androgen receptor have higher anxiety-like behavior than wild-type mice [56, 57]. Our findings here for effects of testosterone on expression of GABA- and BDNF-related genes suggest that the anti-anxiety effects of testosterone reported previously may be mediated by expression of these mood-related genes.

There are limitations of our study that are important to highlight. First, the FCG mice that we conduct our studies with are an artificial system that does not completely match sex-related phenotypes in “normal” wild-type mice. However, the use of the FCG mice are necessary to dissect the sex-related factors that underlie the sex differences observed in wild-type mice and humans. Also, it is possible that testosterone levels may vary by sex chromosome complement during development. Although we do not know that testosterone levels were equivalent at every developmental time point in our mice, we did find that they did not vary by sex chromosome complement at the time of sacrifice for weanlings or adults (see Results). This is especially important to note for the weanling mice, as this confirms that during this organizational window of development, testosterone levels did not vary by sex chromosome complement (see Results). We intentionally selected testosterone as our hormone treatment since it can be converted into estradiol in the brain, and subsequently have action at both estrogen and androgen receptors. However, by doing this, we are not able to discern which of these receptors might be driving the observed effects of testosterone in the current study. We will need to investigate receptor specificity in future studies. As noted earlier, we were also limited given that we did not directly compare our non-stressed and chronically stressed groups within the same experiment. Our use of tissue homogenate of the BLA masks any cell type specific changes that may be occurring. In order to identify these changes, we will have to change tactics moving forward, potentially by using laser microdissection of specific cell types prior to gene expression analyses.

## Conclusions

Our results highlight sex chromosome complement as being an important factor for modulating mood-related genes, especially during pre-pubertal development. Given the observation of organizational effects of hormones in our adult cohorts, which were not found in our pre-pubertal cohorts, our findings suggest that pubertal hormones have an organizing effect on gene expression in the BLA. Additionally, testosterone had different effects in non-stressed vs. chronically stressed conditions based on gonadal sex and, occasionally, sex chromosome complement. Sex differences in the BLA under stressed conditions (especially when compared to the fewer sex differences observed under non-stressed conditions) suggest different molecular profiles in males compared to females with MDD. *SST* modulation in the BLA in particular may be key to understanding an underlying mechanism for anxiety and female vulnerability to MDD. The *Sst* results, in conjunction with our previous behavioral findings, strongly suggest that although there is an underlying vulnerability with XY sex chromosome complement, circulating testosterone usually compensates for this effect in typical males. Without these “protective” levels of circulating testosterone, women might have lower amygdala *SST* expression, resulting in higher MDD vulnerability.

## Abbreviations

ANOVA: Analysis of variance
B: Blank
BDNF: Brain-derived neurotrophic factor
BLA: Basolateral amygdala
DLPFC: Dorsolateral prefrontal cortex
ELISA: Enzyme-linked immunosorbent assay
FCG: Four core genotypes
GABA: Gamma-aminobutyric acid
*Gad67*: Glutamate decarboxylase 67
*Gad65*: Glutamate decarboxylase 65
MDD: Major depressive disorder
qPCR: Quantitative polymerase chain reaction
SEM: Standard error of mean
sgACC: Subgenual anterior cingulate cortex
*Sry*: Sex-determining region Y
*Sst*: Somatostatin
T: Testosterone
*TrkB*: Tropomyosin receptor kinase B
UCMS: Unpredictable chronic mild stress
